# Targeting REV7 effectively reverses 5-FU and oxaliplatin resistance in colorectal cancer

**DOI:** 10.1186/s12935-020-01668-z

**Published:** 2020-12-03

**Authors:** Xianjun Sun, Wenhou Hou, Xin Liu, Jie Chai, Hongliang Guo, Jinming Yu

**Affiliations:** 1 Department of Gastrointestinal Surgery, Shandong Cancer Hospital and Institute, Shandong First Medical University and Shandong Academic Sciences, Jinan, Shandong China; 2 Department of Radiotherapy, Shandong Cancer Hospital and Institute, Shandong First Medical University and Shandong Academic Sciences, 440 Jiyan Rd., Jinan, 250117 Shandong China

**Keywords:** REV7, TLS, 5-FU, Oxaliplatin, Resistance, Colorectal cancer

## Abstract

**Background:**

Despite an enormous research effort, patients diagnosed with advanced colorectal cancer (CRC) still have low prognosis after surgical resection and chemotherapy. The major obstacle for CRC treatment is chemoresistance to front line anti-cancer drugs, such as 5-fluorouracil (5-FU) and oxaliplatin. However, the mechanism of chemoresistance to these drugs remains unclear.

**Methods:**

Cell viability to 5-FU and oxaliplatin was measured by the CellTiter-Glo® 2.0 Cell Viability Assay. The endogenous REV7 protein in CRC cells was detected by western blotting. The translesion synthesis (TLS) events were measured by plasmid-based TLS efficiency assay. Cell apoptosis was evaluated by caspase3/7 activity assay. The in vivo tumor progression was analyzed by HT29 xenograft mice model.

**Results:**

In this study, we found that expression of REV7, which is a key component of translesion synthesis (TLS) polymerase ζ (POL ζ), is significantly increased in both 5-FU and oxaliplatin resistant CRC cells. TLS efficiency analysis revealed that upregulated REV7 protein level results in enhanced TLS in response to 5-FU and oxaliplatin. Importantly, inhibition of REV7 by CRISPR/Cas9 knockout exhibited significant synergy with 5-FU and oxaliplatin in cell culture and murine xenograft model.

**Conclusion:**

These results suggest that combination of REV7 deficiency and 5-FU or oxaliplatin has strong inhibitory effects on CRC cells and identified REV7 as a promising target for chemoresistant CRC treatment.

## Background

Colorectal cancer (CRC) is the third most common cancers worldwide and is often diagnosed at advanced stages [1]. The 5-year survival rate for patients with advanced stage CRC is only 10–15% largely due to resistance to chemotherapy and lack of alternative regimens [2]. Therefore, understanding of mechanisms underlying chemoresistance is extremely important for improving chemotherapy in CRC. 5-fluorouracil (5-FU) has been used as the mainstay of chemotherapy for CRC patients since the 1950s [3]. Other chemotherapy drugs, such as oxaliplatin and irinotecan, have been developed and approved for advanced CRC treatment [4]. 5-FU is a synthetic nucleotide analog that inhibits thymidylate synthase and incorporates its metabolites into DNA, hereby leading to cell death [5]. Oxaliplatin is the third generation-platinum drug that causes cytotoxicity through introducing platinum–DNA adducts [6]. Both 5-FU and oxaliplatin treatments result in disruption of DNA replication. Therefore, tolerance of DNA assault generated by 5-FU or oxaliplatin during replication, which avoids replication folk stalling and subsequent cell death, might related to 5-FU and oxaliplatin resistance.

Replicative DNA polymerases are highly efficient and accurate during DNA synthesis. However, this feature limits their ability to tolerant damaged DNA as a template, thus inhibits progression of the replication fork [7]. Direct replication of damaged DNA can be achieved by translesion synthesis (TLS), a conserved mechanism throughout species from bacteria to mammals [8]. It relies on specialized DNA polymerases with structural feature to accommodate damaged template at the cost of replication fidelity [9]. Genetic studies of lung cancer show that extension TLS DNA polymerase POL ζ (REV3 and REV7) is related to cisplatin-resistance in lung adenocarcinomas [10, 11]. In this study, we found that protein expression of REV7 and TLS efficiency were upregulated in both 5-FU and cisplatin-resistant CRC cells. Genetic inhibition of REV7 significantly improved sensitivity to 5-FU and cisplatin in vivo and in vitro, thereby highlighting the therapeutic potential of inhibiting REV7 in chemoresistant CRC therapy.

## Materials and methods

### Cell culture

HT29 cells (female, colorectal adenocarcinoma cells purchased from ATCC) were grown at 37 °C with 5% CO_2_ in McCoy's 5a medium (SigmaAldrich), 10% (v/v) FBS (Gibco), and 1% Penicillin–Streptomycin antibiotic (Corning). SW480 cells (male, colorectal adenocarcinoma cells purchased from ATCC) were grown at 37 °C with 5% CO2 in L-15 medium (SigmaAldrich), 10% (v/v) FBS (Gibco), and 1% Penicillin–Streptomycin antibiotic (Corning). 5-FU resistant cells were generated by incubating its parental cells with 5-FU at 0.5 µM, 1 µM, 2 µM and 4 µM for 2 weeks (48 h treatment with incubation in drug free medium for 12 days). Oxaliplatin resistant cells were generated by incubating its parental cells with oxaliplatin at 2 µM, 4 µM, 8 µM and 16 µM for 2 weeks (48 h treatment with incubation in drug free medium for 12 days). The resistant cells were kept in the medium with drugs for at least another 16 weeks. Entire population of resistant cells were used in this study.

### Viability assay

Cell viability in response to 5-FU and oxaliplatin was evaluated by the CellTiter-Glo® 2.0 Cell Viability Assay (Promega; G9242) according to the manufacture’s protocol. Briefly, cells were plated at 5000 cells/well in 96-well flat bottom plate (Corning Costar) and incubated for overnight. Increasing doses of 5-FU and oxaliplatin was dissolved in DMSO and added in each well for 48 h. CellTiter-Glo Luminescence stain was added per the manufacturer’s instructions and the luminescence signal was read by the plate reader (BioTek). Relative viability was normalized with DMSO only controls. Transfections were performed using Lipofectamin2000 (ThermoFisher) 24 h before the viability assay according to the manufacture’s protocol.

### Western blotting assay

Cells were washed with ice cold PBS and lysed by ice cold RIPA buffer (20 mM Tris–HCl pH 7.5, 150 mM NaCl, 1 mM Na_2_EDTA, 1 mM EGTA, 1% NP-40, 1% sodium deoxycholate, 2.5 mM sodium pyrophosphate, 1 mM β-glycerophosphate, 1 mM Na_3_VO_4_, 1 µg/ml leupeptin). Cell lysate was harvested and centrifuged at 15,000 rpm for 10 min. Supernatant was collected and its protein concentration was measured using Bradford assay (BioRad) according to the manufactory protocol. Protein samples were loaded and separated on SDS-PAGE gel at 120 V for 50 min and transferred to PVDF membrane at 100 V for 1.5 h. Membranes were subjected to specific antibodies and the protein band intensities were quantitated using ChemiDoc™ XRS + Imaging System (BioRad). Antibodies: REV7 (Abcam; 115622), beta-actin (Cell Signaling Technology; 4967).

### Apoptosis assay

Cell apoptosis assay was performed using caspase3/7 activity kit (Caspase-Glo® 3/7 Assay System; Promega; G8091). Briefly, CRC cells were plated at 3 × 10^5^ cells/ml and incubated for overnight. Cells were treated with 1 µM of 5-FU or 10 µM of oxaliplatin for 24 h. 1 μM of Staurosporine, which activates caspase-3, was employed as positive control. Caspase-3/7 activity was determined according to the manufacture’s protocol.

### Reverse transcription quantitative real-time PCR (RT-qPCR)

The total RNA was extracted by using the RNeasy Mini Kit (Qiagen Corporation) and was reverse transcribed by using the iScript SuperMix reagent (Bio-Rad) according to the manufactures’ instruction. Fluorescence and qPCR were detected by using SYBR Green Super Mix (Bio-Rad) on Real-Time PCR system (CFX Connect; Bio-Rad). The following primers were used: REV7 forward, 5′-GTGGAGAAAGTGGTGGTGGT-3′ and reverse, 5′-TCTTCTCCATGTTGCGAGTG-3′; Actin (intrinsic control) forward, 5′-CACCATTGGCAATGAGCGGTTC-3′ and reverse, 5′-AGGTCTTTGCGGATGTCCACGT-3′

### Xenograft tumor model

1 × 10^7^ HT29-Oxa-R, HT29-Oxa-R-KO, HT29-5-Fu-R or HT29-5-Fu-R-KO cells in 100 µl of PBS were inoculated into the right flank of nude mice (female, 8 weeks old, athymic Foxn1nu; Vital River; Beijing, China). 6 mice were analyzed for each group. Oxaliplatin and 5-Fu was injected every 3 days (oxaliplatin, 3 mg/kg; 5-Fu, 30 mg/kg) when tumor volume reached 100 mm^3^. Tumors volume was calculated using the following formula: (length × width^2^)/2. Tumor volume and body weight were measured every 2 days. Animal experiments were approved and carried out according to the regulations by the Shandong Cancer Hospital and Institute.

### TLS assay

The TLS assay was previous described [12]. Briefly, 200 ng of competitor gapped plasmid and the 50 ng of lesion-containing plasmid were transfected into HT29 and SW480 cells using Lipofectamine2000 (ThermoFisher). Cells were incubated at 37 °C for 4 h and harvested. Subsequently, DNA was extracted using DNA isolation kit (QIAGEN) and transformed into the recA- E. coli to propagate closed plasmids for 16 h. Plasmid DNA was extracted from E. coli culture and lesion region was amplified by PCR (forward primer: 5′ TTGTACTGAGAGTGCACCATGCCCGT-3′, reverse primer: 5′-GAGTCAGTGAGCGAGGAAGCGTGCTG-3′). Restriction enzymes XhoI and SphI (NEB) were used to digest the PCR products, which were next subjected to TSQ Altis™ Triple Quadrupole Mass Spectrometer (ThermoFisher) to determine the nucleotides originally repaired in CRC cells.

### CRISPR/Cas9 knockout

Guide RNA sequences targeting REV7 were cloned into the pSpCas9(BB)-2A-GFP (PX458) vector, a gift from Feng Zhang (Addgene; 48138; Massachusetts, USA.) [13]. The plasmid was transfected in 5-FU and oxaliplatin resistant CRC cells using Lipofectamine2000 (ThermoFisher) and incubated for 24 h. GFP positive cells, which indicating successful transfection, were selected and seeded using BD FACSMelody cell sorter (BD biosciences). Single clones were harvested after 14 days and REV7 deficiency was validated using western blotting assay.

### Statistical analysis

Statistical analysis was performed using Prism7 software (GraphPad). Student’s t-test was used for comparison of two groups. One-way ANOVA analysis with a Bonferroni post-test was used to compare multiple groups. *p < 0.05 was considered significant. Variation is indicated and presented as mean ± SEM.

## Results

### Establishment of chemoresistant CRC cell lines

**Fig. 1:**
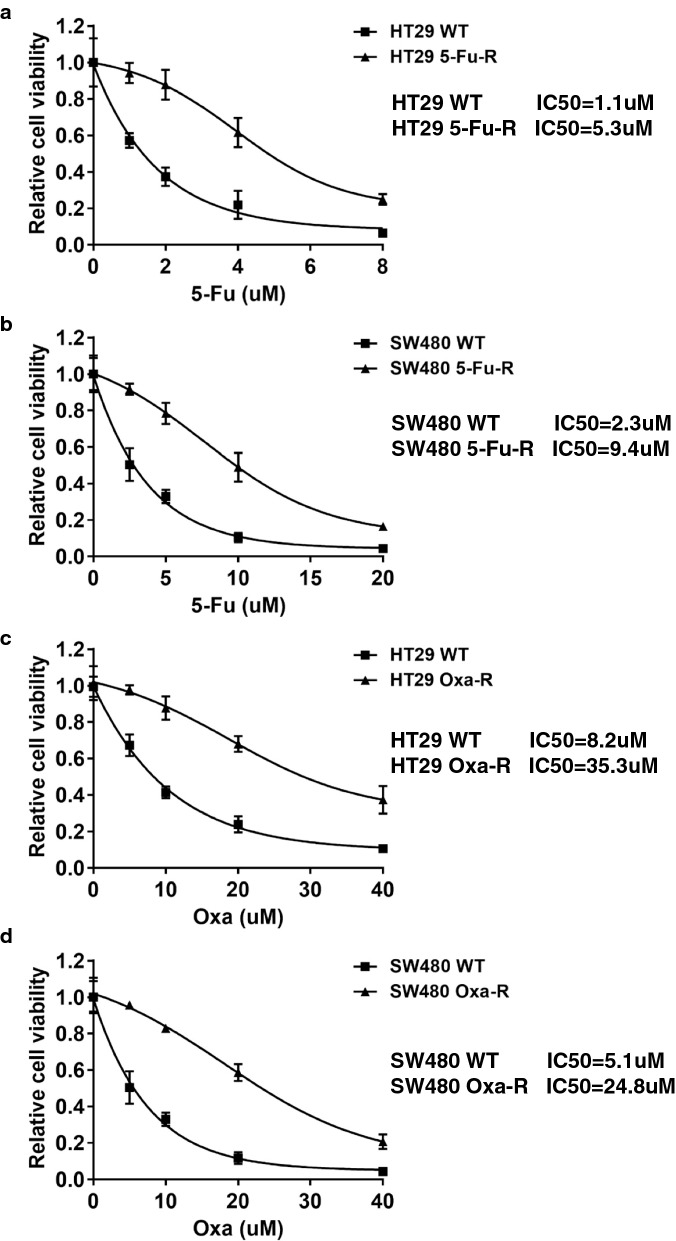
Generation of 5-Fu and oxaliplatin resistant CRC cell lines. **a** Relative cell viability to 5-Fu in HT29 WT and HT29 5-Fu-R cell lines. Cells were treated with 0 µM, 1 µM, 2 µM, 4 µM, 8 µM of 5-Fu for 48 h. **b** Relative cell viability to 5-Fu in SW480 WT and SW480 5-Fu-R cell lines. Cells were treated with 0 µM, 2.5 µM, 5 µM, 10 µM, 20 µM of 5-Fu for 48 h. **c** Relative cell viability to oxaliplatin in HT29 WT, HT29 5-Oxa-R, **d** SW480 WT and SW480 Oxa-R cell lines. Cells were treated with 0 µM, 5 µM, 10 µM, 20 µM, 40 µM of oxalipatin for 48 h. The statistical analysis of cell viability was calculated by using one-way ANOVA. Variation is indicated and presented as mean ± SEM

Previous studies show that TLS polymerases, such as REV1 and Polζ (REV3 and REV7), are positively correlated with cisplatin resistance [14]. Since TLS is the common pathways triggered by both 5-FU and oxaliplatin, we hypothesize that TLS polymerases play a role in mechanism of 5-FU and oxaliplatin resistance. To evaluate TLS factors that might contribute to resistance mechanism in CRC, we first generated 5-FU and oxaliplatin resistant cell lines. HT29 and SW480 cells were repeatedly exposed to a range of drug concentrations in cell culture for 2 months. To the end, the sensitivity of paired cell lines was determined by cell viability to 5-FU and oxaliplatin. As shown in Fig. 1a, b, 5-FU resistant HT29 (HT29 5-FU-R) and 5-FU resistant SW480 (SW480 5-FU-R) cells showed a 4.82-fold and 4.09-fold increase in resistance to 5-FU respectively, based on a comparison of IC50 values. In addition, oxaliplatin resistant HT29 (HT29 Oxa-R) and oxaliplatin resistant SW480 (SW480 Oxa-R) exhibited 4 to fivefold higher IC50 as compared to their parental cell lines (Fig. 1c, d).

### REV7 is upregulated in 5-FU and oxaliplatin resistant CRC cells

**Fig. 2:**
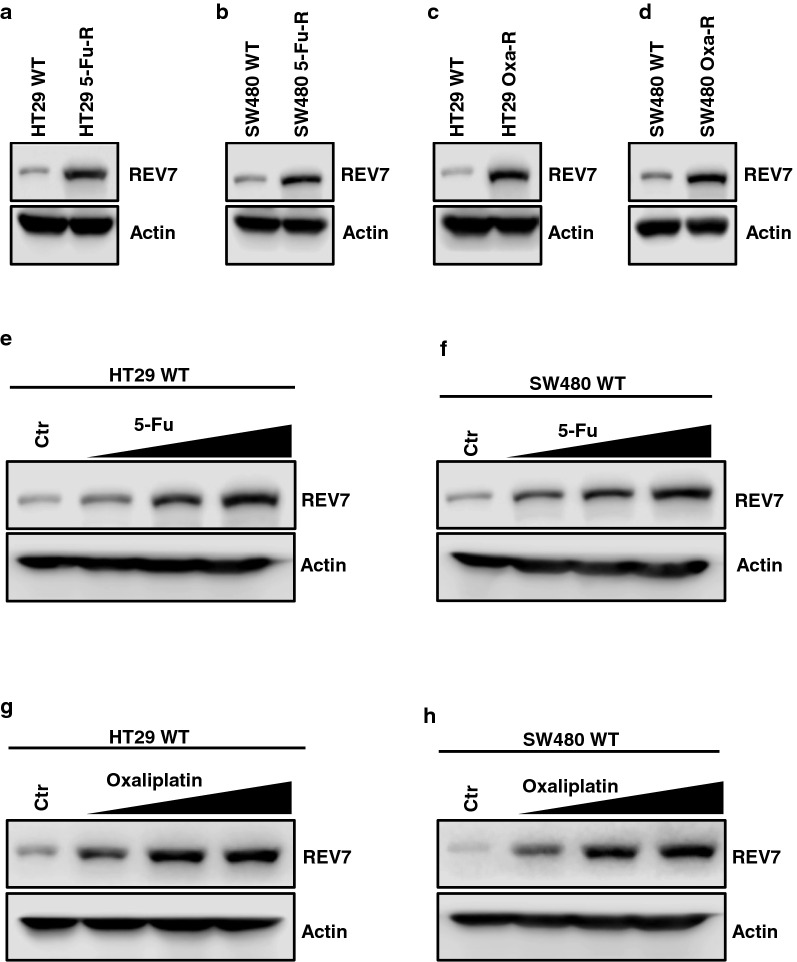
REV7 is upregulated in 5-FU and oxaliplatin resistant CRC cells. **a** Western blot analysis of REV7 protein expression in HT29 WT, HT29 5-Fu-R, **b** SW480 WT, SW480 5-Fu-R, **c** HT29 Oxa-R and **d** SW480 Oxa-R cells**.** Expression of β-actin (Actin) was used as internal control. **e** Western blot analysis of REV7 protein expression in response to 5-Fu in HT29 WT and **f** SW480 WT cells. Cells were treated with 0 µM, 0.5 µM, 1 µM and 2 µM of 5-Fu for 48 h before harvesting for western blotting analysis. **g** Western blot analysis of REV7 protein expression in response to oxaliplatin in HT29 WT and **h** SW480 WT cells. Cells were treated with 0 µM, 2 µM, 4 µM and 8 µM of oxaliplatin for 48 h before harvesting for western blotting analysis

Using western blotting assay, we identified that REV7, which is the scaffolding protein tethering REV3 and REV1, was upregulated in both 5-FU and oxaliplatin resistant CRC cells (Fig. 2a–d), indicating that REV7 participates in the common pathway shared by mechanisms underlying 5-FU and oxaliplatin resistance. To provide direct evidence that REV7 expression is induced by 5-FU and oxaliplatin, we treated the CRC wild type (WT) cells with 5-FU and oxaliplatin for 3 days and observed that REV7 expression was increased in a dose dependent manner (Fig. 2e–h).

To investigate whether 5-Fu and oxaliplatin induce REV7 at transcriptional level, we evaluated REV7 mRNA expression in response to 5-Fu or oxaliplatin. As shown in Additional file 1: Figure S1A–D, 5-Fu and oxaliplatin did not significantly affect REV7 mRNA expression, suggesting that the increased REV7 protein is not caused by induced transcription. We also measured REV7 protein expression after drug treatment in the presence of protein synthesis inhibitor cycloheximide (CHX). We found that REV7 expression failed to be induced by 5-Fu or oxaliplatin, indicating that 5-Fu and oxaliplatin translationally increased REV7 expression (Additional file 1: Figure S2A–D). In addition, in the presence of proteasome inhibitor MG132, REV7 protein expression was still induced by 5-Fu and oxaliplatin (Additional file 1: Figure S3A–D), indicating the elevated REV7 is not due to decrease of proteasome-mediated degradation.

These results suggest that REV7 indeed regulates 5-FU and oxaliplatin resistance in CRC.

### TLS efficiency is increased in 5-FU and oxaliplatin resistant CRC cells

**Fig. 3:**
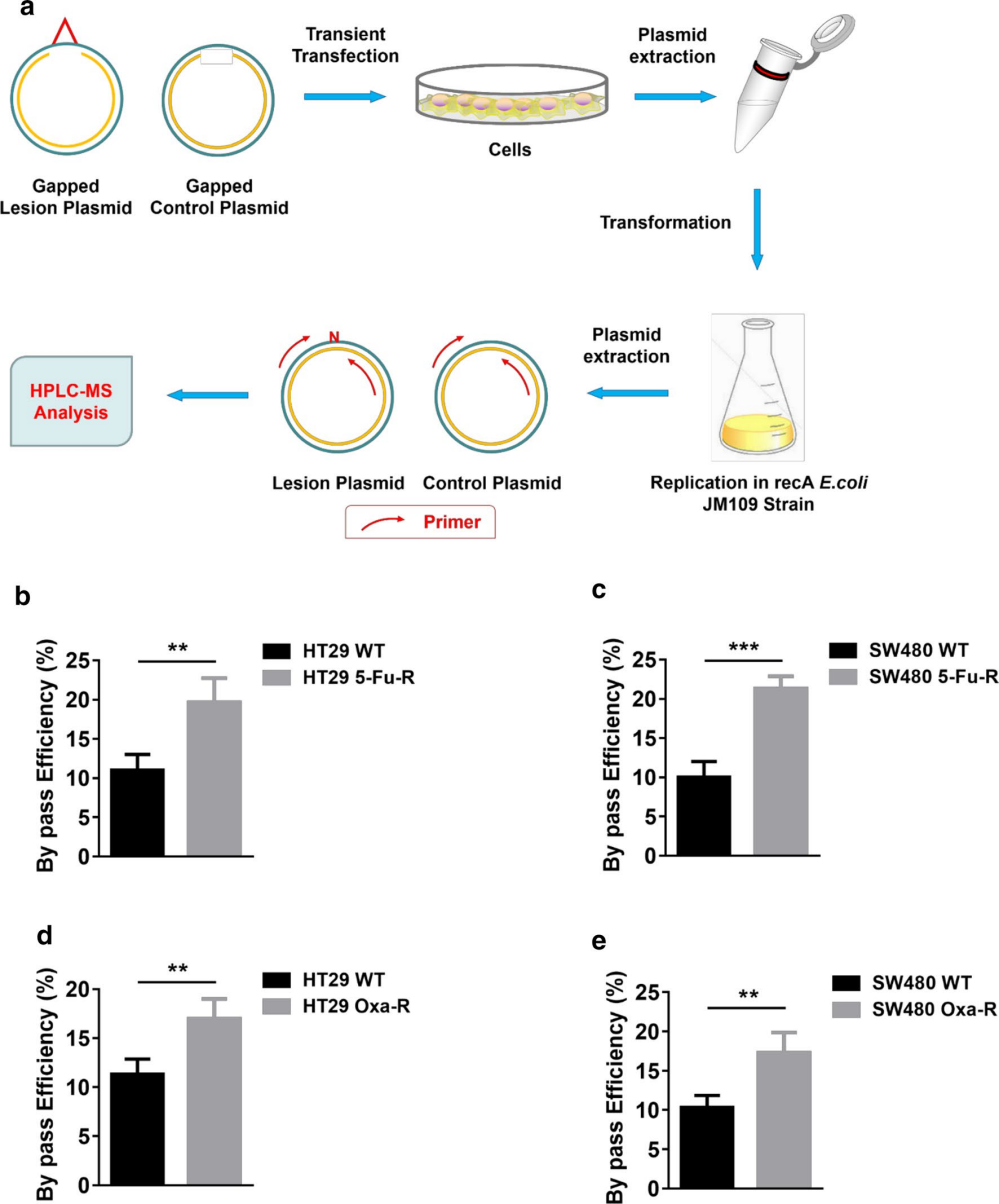
TLS efficiency is increased in 5-FU and oxaliplatin resistant CRC cells. **a** Outline of plasmid-based TLS efficiency assay. Procedures was described in “Material and methods” section. **b** Plasmid-based TLS efficiency analysis in HT29 WT, HT29 5-Fu-R, **c** SW480 WT, SW480 5-Fu-R, **d** HT29 Oxa-R and **e** SW480 Oxa-R. **p < 0.01; ***p < 0.001. The p-values were calculated by using unpaired two-tailed Student's t-test. Variation is indicated and presented as mean ± SEM

REV7 is essential for efficient TLS in mammalian cells [15, 16]. Therefore, we then determined whether TLS efficiency is altered in 5-FU and oxaliplatin resistant CRC cells using a plasmid-based TLS efficiency assay [12]. Gapped lesion plasmid, which mimics damaged template in replication, and gapped control plasmid were transfected in WT and resistant CRC cells to allow TLS. The closed plasmids were propagated in *E. coli* and the gap region was amplified by PCR followed by restriction enzyme digestion. The final nucleotides were determined by LC–MS (Fig. 3a). As we expected, bypass efficiency of 5-FU resistant HT29 and SW480 cells were significantly higher than that of WT cell lines (Fig. 3b, c). Similar results were found in oxaliplatin resistant CRC cells (Fig. 3d, e), indicating upregulated TLS contributes to CRC cell resistance to 5-FU and oxaliplatin.

### REV7 deficiency reduces TLS efficiency in 5-FU and oxaliplatin resistant CRC cells

**Fig. 4:**
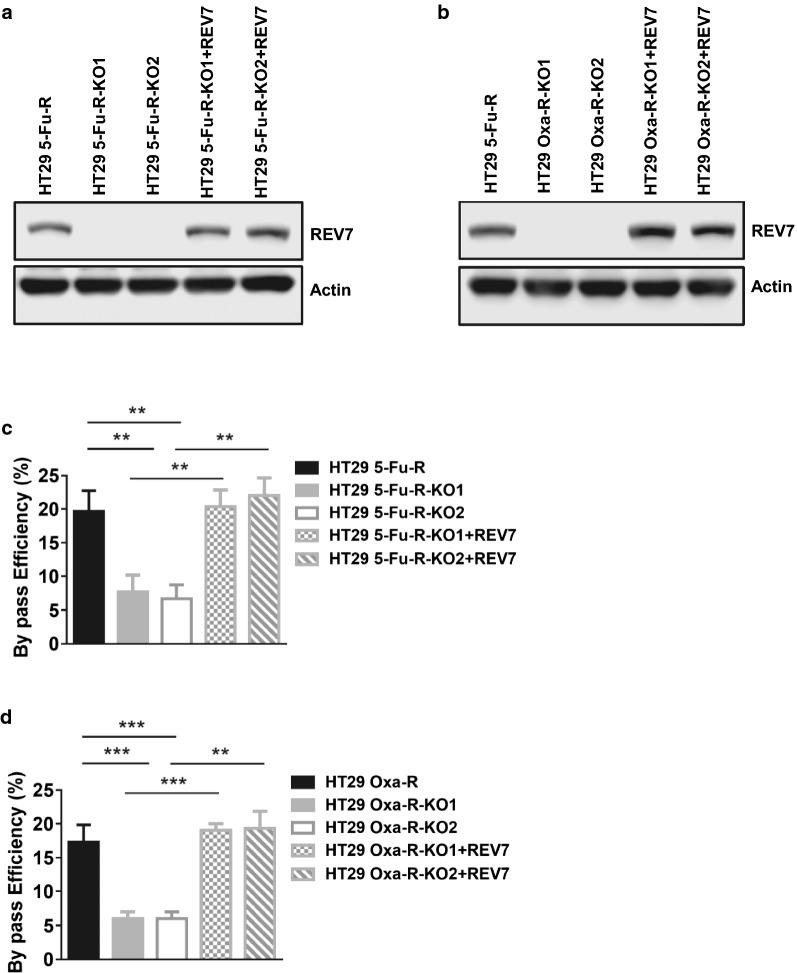
REV7 deficiency reduces TLS efficiency in 5-FU and oxaliplatin resistant CRC cells. **a** Western blot analysis of REV7 protein expression in HT29 5-Fu-R, REV7 deficient HT29 5-Fu-R clone 1 (HT29 5-Fu-R-KO1), REV7 deficient HT29 5-Fu-R clone 2 (HT29 5-Fu-R-KO2), HT29 5-Fu-R-KO1 with complemented REV7 (HT29 5-Fu-R-KO1 + REV7) and HT29 5-Fu-R-KO2 with complemented REV7 cell lines (HT29 5-Fu-R-KO2 + REV7). **b** Western blot analysis of REV7 protein expression in HT29 Oxa-R, REV7 deficient HT29 Oxa-R clone 1 (HT29 Oxa-R-KO1), REV7 deficient HT29 Oxa-R clone 2 (HT29 Oxa-R-KO2), HT29 Oxa-R-KO1 with complemented REV7 (HT29 Oxa-R-KO1 + REV7) and HT29 Oxa-R-KO2 with complemented REV7 cell lines (HT29 Oxa-R-KO2 + REV7). **c** Plasmid-based TLS efficiency analysis in cell lines from Fig. 3a and **d** Fig. 3b. **p < 0.01; ***p < 0.001 The p-values were calculated by using. Variation is indicated and presented as mean ± SEM

Given that REV7 and TLS efficiency were increased in response to 5-FU and oxaliplatin, we then asked whether elevated TLS in resistant CRC cells is caused by upregulated REV7. Using CRISPR/Cas9 targeting REV7, we generated 2 clones of REV7 deficient 5-FU and oxaliplatin resistant HT29 cells (HT29 5-FU-R-KO1, HT29 5-FU-R-KO2, HT29 Oxa-R-KO1, HT29 Oxa-R-KO2,) (Fig. 4a, b). We then measured TLS efficiency in the absence and presence of REV7 and found that REV7 deficiency significantly inhibited TLS in both 5-FU and oxaliplatin resistant HT29 cells (Fig. 4c, d). To eliminate the off-target effects of CRISPR/Cas9 that might affect the TLS assay, we measured TLS efficiency in REV7 deficient HT29 cells complemented with WT REV7. As shown in Fig. 4a–d, REV7 expression in REV7 deficient cells rescued TLS efficiency, suggesting that REV7 indeed regulates TLS in 5-FU and oxaliplatin resistant CRC cells.

### *Inhibition of REV7 overcomes resistance to 5-FU and oxaliplatin *in vitro

**Fig. 5:**
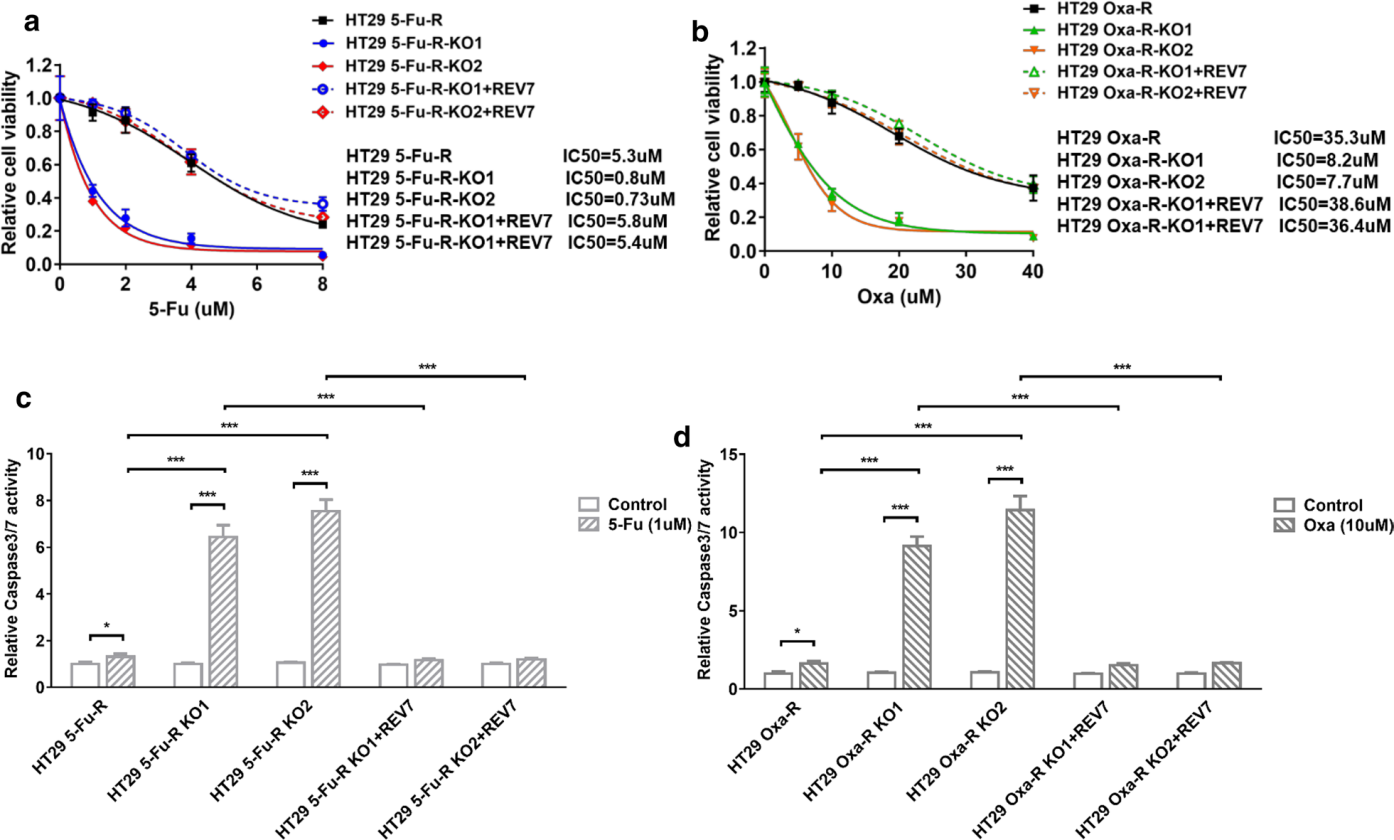
Inhibition of REV7 overcomes resistance to 5-FU and oxaliplatin in vitro. **a** Relative cell viability to 5-Fu in HT29 5-Fu-R, HT29 5-Fu-R-KO1, HT29 5-Fu-R-KO2, HT29 5-Fu-R-KO1 + REV7 and HT29 5-Fu-R-KO2 + REV7 cell lines. Cells were treated with 0 µM, 1 µM, 2 µM, 4 µM, 8 µM of 5-Fu for 48 h. The statistical analysis of cell viability was calculated by using one-way ANOVA. Variation is indicated and presented as mean ± SEM. **b** Relative cell viability to oxaliplatin in HT29 Oxa-R, HT29 Oxa-R-KO1, HT29 Oxa-R-KO2, HT29 Oxa-R-KO1 + REV7 and HT29 Oxa-R-KO2 + REV7 cell lines. Cells were treated with 0 µM, 5 µM, 10 µM, 20 µM, 40 µM of oxaliplatin for 48 h. The statistical analysis of cell viability was calculated by using one-way ANOVA. Variation is indicated and presented as mean ± SEM. **c** 5-Fu (1 µM) induced relative caspase 3/7 activity in cells analyzed in Fig. 4a. **d** Oxaliplatin (10 µM) induced relative caspase 3/7 activity in cells analyzed in Fig. 4b. *p < 0.05; ***p < 0.001. The p-values were calculated by using by one-way ANOVA. Variation is indicated and presented as mean ± SEM

To determine whether REV7 deficiency sensitize CRC cells to 5-FU, we compared cell viability in response to 5-FU between REV7 proficient and deficient HT29 5-FU-R cells. As shown in Fig. 5a, deletion of REV7 improved cell sensitivity to 5-FU by 7 folds and REV7 complementation restored 5-FU resistance. Similar observation was also found in HT29 Oxa-R cells in response to oxaliplatin (Fig. 5b), suggesting that REV7 is a potential target for 5-FU and oxaliplatin resistant CRC patients. Using caspase 3/7 activity assay, we also found that REV7 deficiency profoundly increased caspase 3/7 activity in response to 5-FU and oxaliplatin (Fig. 5c, d), indicating that combination of REV7 deficiency and 5-FU or oxaliplatin generates synergy through upregulated cell apoptosis.

### *REV7-deficiency inhibited oxaliplatin-resistant HT29 xenograft tumor growth *in vivo

**Fig. 6:**
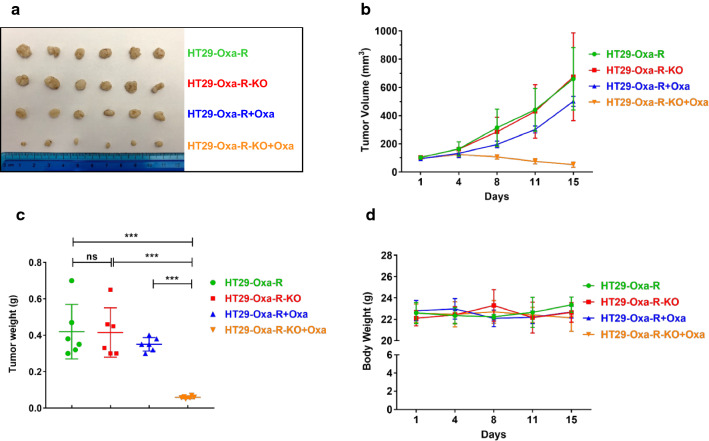
REV7-deficiency inhibited oxaliplatin-resistant HT29 xenograft tumor growth in vivo. **a** Photograph of inoculated tumors excised at day 15. HT29 Oxa-R and HT29 Oxa-R-KO tumors with or without oxaliplatin treatment were evaluated in this analysis. **b** Growth curve of inoculated tumors. Variation is indicated and presented as mean ± SEM. **c** Weight of inoculated tumors excised at day 15. ns: not significant; ***p < 0.001. The p-values were calculated by using by one-way ANOVA. Variation is indicated and presented as mean ± SEM. **d** Body weight of the mice measured post-drug treatment. No significant variation was detected. The statistical analysis of cell viability was calculated by using two-way ANOVA

We further demonstrated tumor-suppressive synergy between REV7 deficiency and oxaliplatin in vivo*.* We first established murine xenograft model. HT29 Oxa-R and HT29 Oxa-R-KO cells were subcutaneously inoculated into lower flanks in nude mice (female, 8 weeks old, athymic Foxn1nu, 6 mice/group). Oxaliplatin or vehicle was given when tumor volume reached 100 mm^3^. As shown in Fig. 6a–c, expression of REV7 did not affect tumor volume without oxaliplatin treatment, indicating REV7 does not affect tumorigenesis in CRC. Notably, oxaliplatin exhibited excellent tumor growth suppression in HT29 Oxa-R-KO xenograft as compared to REV7 proficient HT29 Oxa-R xenograft with no significant difference in body weight (Fig. 6a–d). To determine whether REV7 contributes to 5-Fu resistance in vivo, we used HT29 5-Fu-R and HT29 5-Fu-R-KO xenograft to evaluate tumor-suppressive synergy between REV7 deficiency and 5-Fu. As shown in Additional file 1: Figure S4A–C, REV7 deficient xenograft exhibited significant sensitivity to 5-Fu as compared to REV7 proficient xenograft without obvious toxicity. Therefore, we confirmed that inhibition of REV7 generates synergy with front-line chemotherapy in CRC in vivo.

## Discussion

**Fig. 7:**
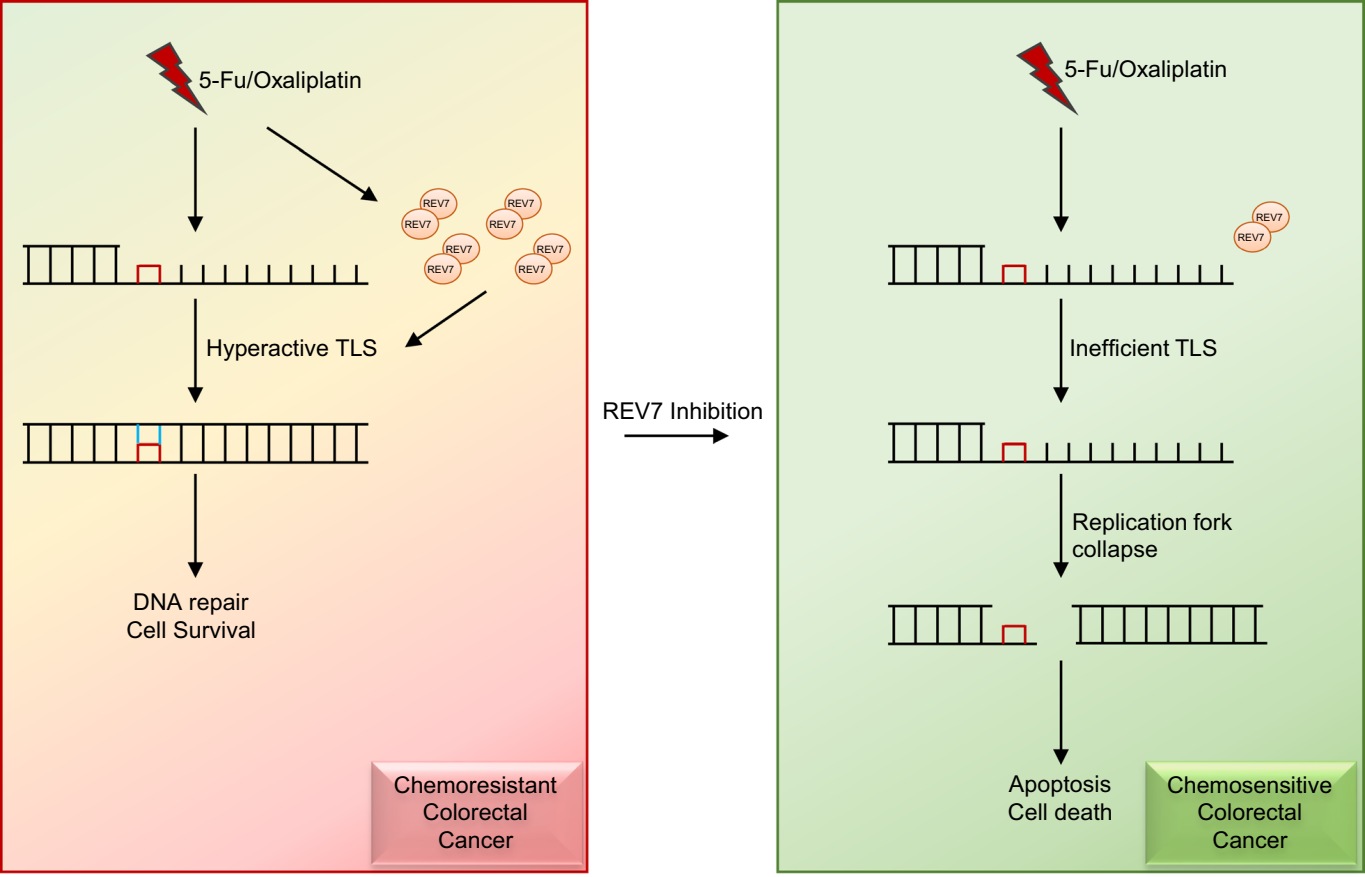
Model for targeting REV7 in 5-FU and oxaliplatin resistant colorectal cancer

5-FU has been widely used as anticancer drug since 1957 and contributed in the treatment of variety types of cancer, such as CRC, breast cancer and head and neck cancer [17]. Despite many advantages provided by 5-FU, its clinical efficacy has been largely limited due to drug resistance. Upregulated thymidylate synthase expression is commonly acknowledged as its major molecular mechanism for 5-FU resistance [18]. However, Contributions from multiple factors and pathways have been recognized, including methylation of the *MLH1, o*verexpression of anti-apoptosis proteins, dysregulated DNA repair [19–22]. Our study, for the first time, identified that TLS play an important role in 5-FU resistance. Significantly, both 5-FU and oxaliplatin resistant CRC cells exhibited elevated expression of REV7. Although 5-FU and oxaliplatin generate DNA damage through different mechanisms, they all trigger upregulation of TLS via inducing REV7 expression, suggesting a novel DNA repair mechanism in accordance with DNA damage induced by these drugs and a very promising role of REV7 as a target to overcome 5-FU and oxaliplatin resistance in CRC (Fig. 7).

Obtaining specific small molecule targeting TLS is challenging because replicative and TLS polymerase share same substrate and loading proteins [23, 24]. Hashimoto et al. found that evolutionarily conserved interaction between insertion TLS polymerase REV1 and REV7 is specific and critical to efficient mutagenic TLS [25]. This research led to a recent discovery of a potent REV1-REV7 interface inhibitor, JH-RE-06, which exhibited TLS inhibition and significant synergy with cisplatin in human fibrosarcoma, melanoma and prostate adenocarcinoma cells [12]. Together, these findings and our observations render the REV1-REV7 interface an outstanding target for development of combination chemotherapies with 5-FU and oxaliplatin in CRC.

## Conclusion

Taken together, we found that TLS key factor REV7 was overexpressed in 5-FU and oxaliplatin resistant CRC cells. Consequently, elevated expression of REV7 results in increase of TLS efficiency and resistance to 5-FU and oxaliplatin. REV7 deficiency exhibited excellent synergy with chemotherapy drugs in vitro and in vivo. Our results suggest that targeting REV7 is a promising approach to overcome chemoresistance in CRC patients.

## Supplementary Information

**Additional file 1: Figure S1.** mRNA of REV7 is not induced in response to 5-FU and oxaliplatin. **Figure S2.** Increase of REV7 in response to 5-FU and oxaliplatin is mediated by protein sysnthesis. **Figure S3.** Increase of REV7 in response to 5-FU and oxaliplatin is not mediated by proteasome-mediated degradation. **Figure S4.** REV7-deficiency inhibited 5-Fu-resistant HT29 xenograft tumor growth in vivo.

## Data Availability

All data generated or analyzed during this study are included in this published article.

## References

[1] Siegel RL, Miller KD, Fedewa SA, Ahnen DJ, Meester RGS, Barzi A, Jemal A (2017). Colorectal cancer statistics. CA Cancer J Clin.

[2] Brenner H, Kloor M, Pox CP (2014). Colorectal cancer. Lancet.

[3] Salonga D, Danenberg KD, Johnson M, Metzger R, Groshen S, Tsao-Wei DD, Lenz HJ, Leichman CG, Leichman L, Diasio RB, Danenberg PV (2000). Colorectal tumors responding to 5-fluorouracil have low gene expression levels of dihydropyrimidine dehydrogenase, thymidylate synthase, and thymidine phosphorylase. Clin Cancer Res.

[4] Yaffee P, Osipov A, Tan C, Tuli R, Hendifar A (2015). Review of systemic therapies for locally advanced and metastatic rectal cancer. J Gastrointest Oncol.

[5] Longley DB, Harkin DP, Johnston PG (2003). 5-fluorouracil: mechanisms of action and clinical strategies. Nat Rev Cancer.

[6] Martinez-Balibrea E, Martinez-Cardus A, Gines A, Ruiz de Porras V, Moutinho C, Layos L, Manzano JL, Buges C, Bystrup S, Esteller M, Abad A (2015). Tumor-related molecular mechanisms of oxaliplatin resistance. Mol Cancer Ther.

[7] Johansson E, Dixon N (2013). Replicative DNA polymerases. Cold Spring Harb Perspect Biol.

[8] Sale JE (2013). Translesion DNA synthesis and mutagenesis in eukaryotes. Cold Spring Harb Perspect Biol.

[9] Vaisman A, Woodgate R (2017). Translesion DNA polymerases in eukaryotes: what makes them tick?. Crit Rev Biochem Mol Biol.

[10] Doles J, Oliver TG, Cameron ER, Hsu G, Jacks T, Walker GC, Hemann MT (2010). Suppression of Rev3, the catalytic subunit of Pol{zeta}, sensitizes drug-resistant lung tumors to chemotherapy. Proc Natl Acad Sci U S A.

[11] Xie K, Doles J, Hemann MT, Walker GC (2010). Error-prone translesion synthesis mediates acquired chemoresistance. Proc Natl Acad Sci U S A.

[12] Wojtaszek JL, Chatterjee N, Najeeb J, Ramos A, Lee M, Bian K, Xue JY, Fenton BA, Park H, Li D, Hemann MT, Hong J, Walker GC, Zhou P (2019). A Small molecule targeting mutagenic translesion synthesis improves chemotherapy. Cell.

[13] Bluteau D, Masliah-Planchon J, Clairmont C, Rousseau A, Ceccaldi R, Dubois d'Enghien C, Bluteau O, Cuccuini W, Gachet S, Peffault de Latour R, Leblanc T, Socie G, Baruchel A, Stoppa-Lyonnet D, D'Andrea AD, Soulier J (2016). Biallelic inactivation of REV7 is associated with Fanconi anemia. J Clin Invest.

[14] Sharma S, Shah NA, Joiner AM, Roberts KH, Canman CE (2012). DNA polymerase zeta is a major determinant of resistance to platinum-based chemotherapeutic agents. Mol Pharmacol.

[15] Takezawa J, Ishimi Y, Aiba N, Yamada K (2010). Rev1, Rev3, or Rev7 siRNA abolishes ultraviolet light-induced translesion replication in hela cells: a comprehensive study using alkaline sucrose density gradient sedimentation. J Nucleic Acids.

[16] Hicks JK, Chute CL, Paulsen MT, Ragland RL, Howlett NG, Gueranger Q, Glover TW, Canman CE (2010). Differential roles for DNA polymerases eta, zeta, and REV1 in lesion bypass of intrastrand versus interstrand DNA cross-links. Mol Cell Biol.

[17] Grem JL (2000). 5-Fluorouracil: forty-plus and still ticking. A review of its preclinical and clinical development. Invest New Drugs.

[18] Yoshioka A, Tanaka S, Hiraoka O, Koyama Y, Hirota Y, Ayusawa D, Seno T, Garrett C, Wataya Y (1987). Deoxyribonucleoside triphosphate imbalance. 5-Fluorodeoxyuridine-induced DNA double strand breaks in mouse FM3A cells and the mechanism of cell death. J Biol Chem.

[19] Arnold CN, Goel A, Boland CR (2003). Role of hMLH1 promoter hypermethylation in drug resistance to 5-fluorouracil in colorectal cancer cell lines. Int J Cancer.

[20] Liu R, Page C, Beidler DR, Wicha MS, Nunez G (1999). Overexpression of Bcl-x(L) promotes chemotherapy resistance of mammary tumors in a syngeneic mouse model. Am J Pathol.

[21] Violette S, Poulain L, Dussaulx E, Pepin D, Faussat AM, Chambaz J, Lacorte JM, Staedel C, Lesuffleur T (2002). Resistance of colon cancer cells to long-term 5-fluorouracil exposure is correlated to the relative level of Bcl-2 and Bcl-X(L) in addition to Bax and p53 status. Int J Cancer.

[22] Wyatt MD, Wilson DM (2009). Participation of DNA repair in the response to 5-fluorouracil. Cell Mol Life Sci.

[23] Bhat A, Wu Z, Maher VM, McCormick JJ, Xiao W (2015). Rev7/Mad2B plays a critical role in the assembly of a functional mitotic spindle. Cell Cycle.

[24] Boersma V, Moatti N, Segura-Bayona S, Peuscher MH, van der Torre J, Wevers BA, Orthwein A, Durocher D, Jacobs JJL (2015). MAD2L2 controls DNA repair at telomeres and DNA breaks by inhibiting 5' end resection. Nature.

[25] Hashimoto K, Cho Y, Yang IY, Akagi J, Ohashi E, Tateishi S, de Wind N, Hanaoka F, Ohmori H, Moriya M (2012). The vital role of polymerase zeta and REV1 in mutagenic, but not correct, DNA synthesis across benzo[a]pyrene-dG and recruitment of polymerase zeta by REV1 to replication-stalled site. J Biol Chem.

