# Supplementary material for: Targeting REV7 effectively reverses 5-FU and oxaliplatin resistance in colorectal cancer
Source: Cancer Cell Int. 2020 Dec 3;20:580. doi: 10.1186/s12935-020-01668-z (PMC7713438; doi:10.1186/s12935-020-01668-z)
Supplement: Supplementary file 1 — Additional file 1: Figure S1. mRNA of REV7 is not induced in response to 5-FU and oxaliplatin. Figure S2. Increase of REV7 in response to 5-FU and oxaliplatin is mediated by protein sysnthesis. Figure S3. Increase of REV7 in response to 5-FU and oxaliplatin is not mediated by proteasome-mediated degradation. Figure S4. REV7-deficiency inhibited 5-Fu-resistant HT29 xenograft tumor growth in vivo. [file 12935_2020_1668_MOESM1_ESM.docx]

**Additional data**

**Targeting REV7 effectively reverses 5-Fu and oxaliplatin resistance in colorectal cancer**

Xianjun Sun, Wenhou Hou, Xin Liu, Jie Chai, Hongliang Guo, Jinming Yu

This file includes additional Figures S1 to S4


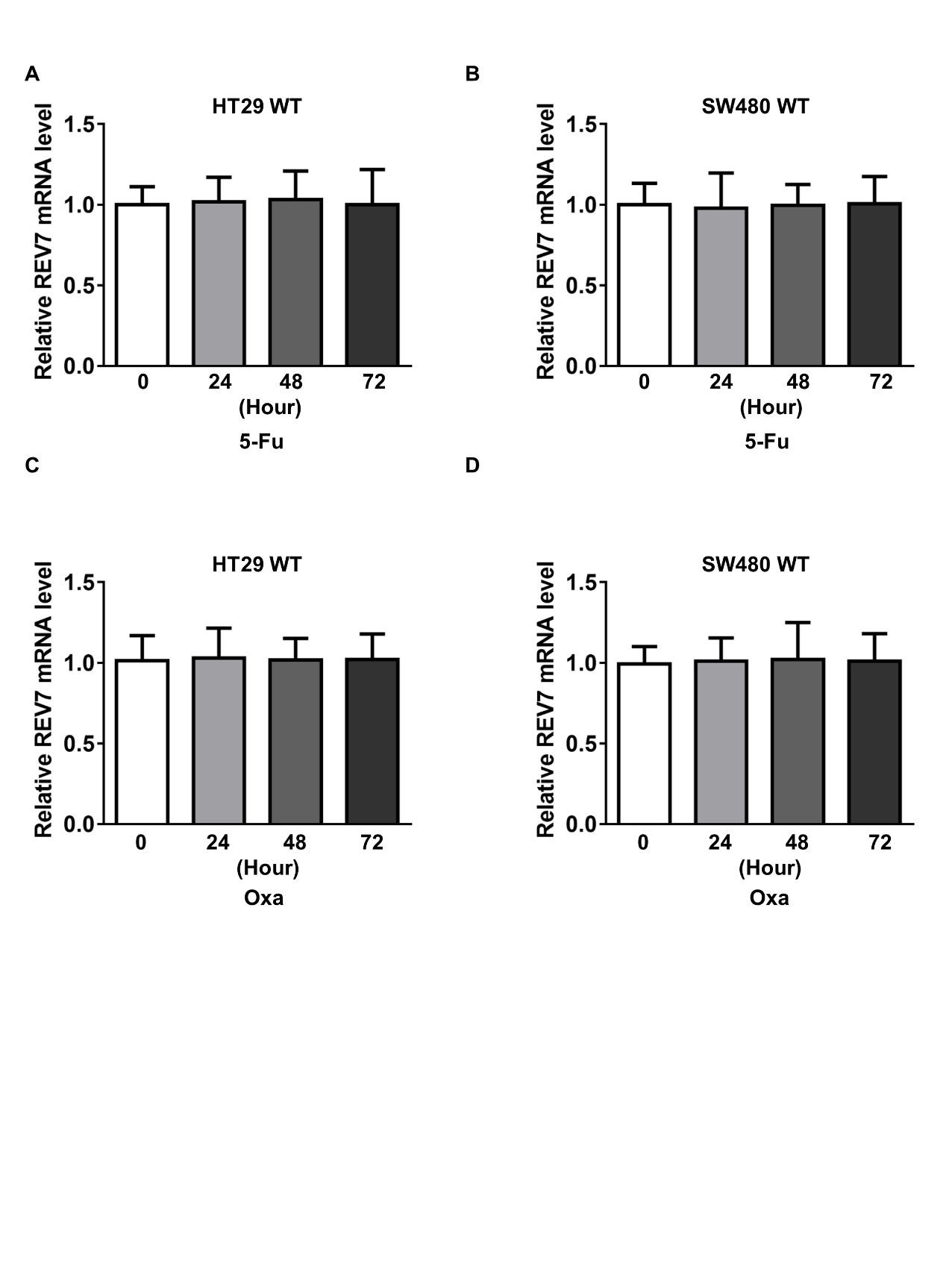


**Figure. S1 mRNA of REV7 is not induced in response to 5-FU and oxaliplatin.**

**(A)** Expression of REV7 mRNA in response to 5-Fu in HT29 WT and **(B)** SW480 WT cells. Cells were treated with 1 µM of 5-Fu for 0 h, 24 h, 48 h and 72 h before harvesting for RT-qPCR analysis. **(C)** Expression of REV7 mRNA in response to oxaliplatin in HT29 WT and **(D)** SW480 WT cells. Cells were treated with 4 µM of oxaliplatin for 0 h, 24 h, 48 h and 72 h before harvesting for RT-qPCR analysis.


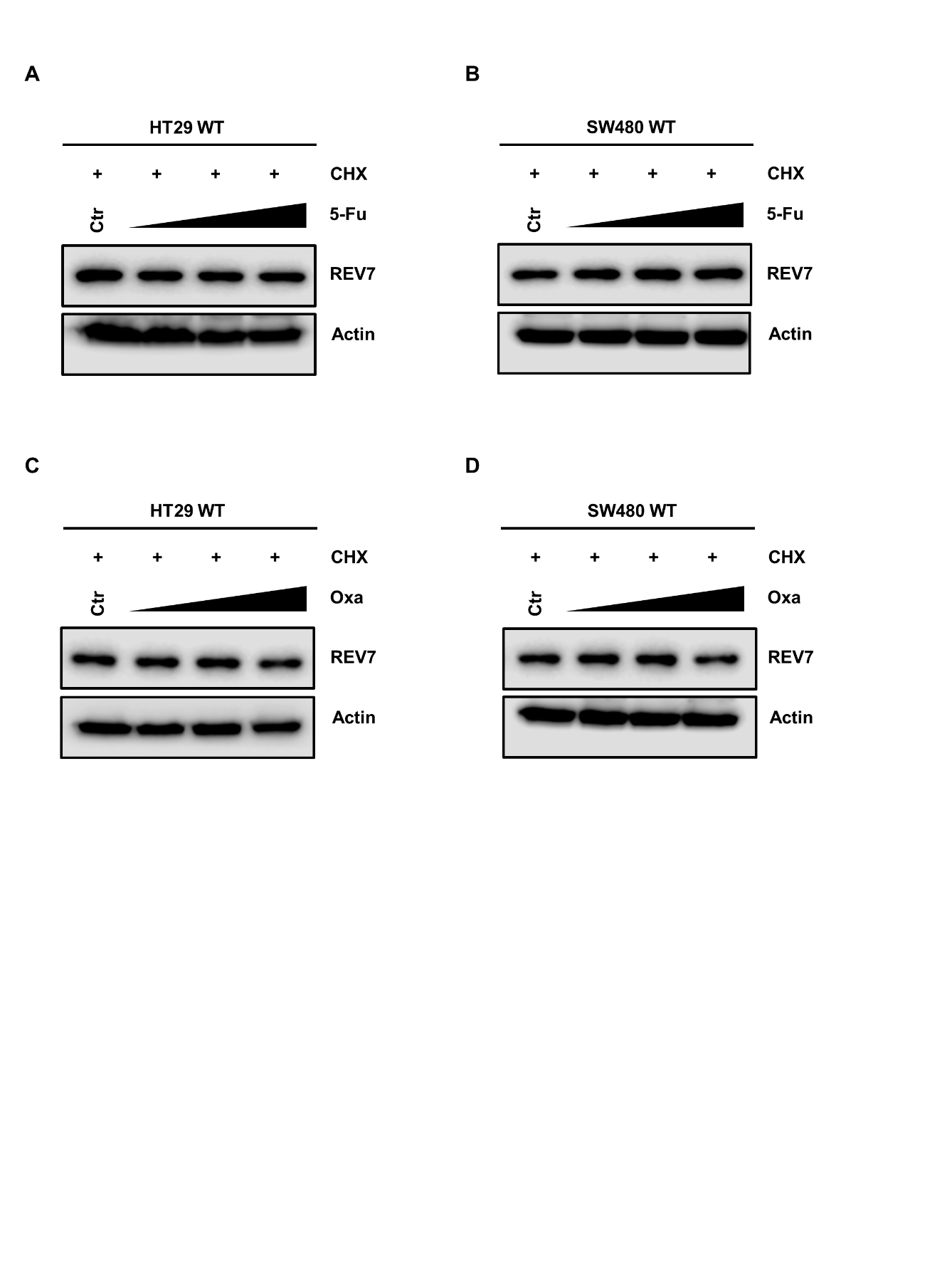


**Figure. S2 Increase of REV7 in response to 5-FU and oxaliplatin is mediated by protein sysnthesis**

**(A)** Western blot analysis of REV7 protein expression in response to 5-Fu in HT29 WT and **(B)** SW480 WT cells in the presence of cycloheximide (CHX). Cells were subjected to 30 μg/ml cycloheximide and subsequently treated with 0 µM, 0.5 µM, 1 µM and 2 µM of 5-Fu for 48 h before harvesting for western blotting analysis. **(C)** Western blot analysis of REV7 protein expression in response to oxaliplatin in HT29 WT and (**D)** SW480 WT cells. Cells were subjected to 30 μg/ml CHX and subsequently treated with 0 µM, 2 µM, 4 µM and 8 µM of oxaliplatin for 48 h before harvesting for western blotting analysis.


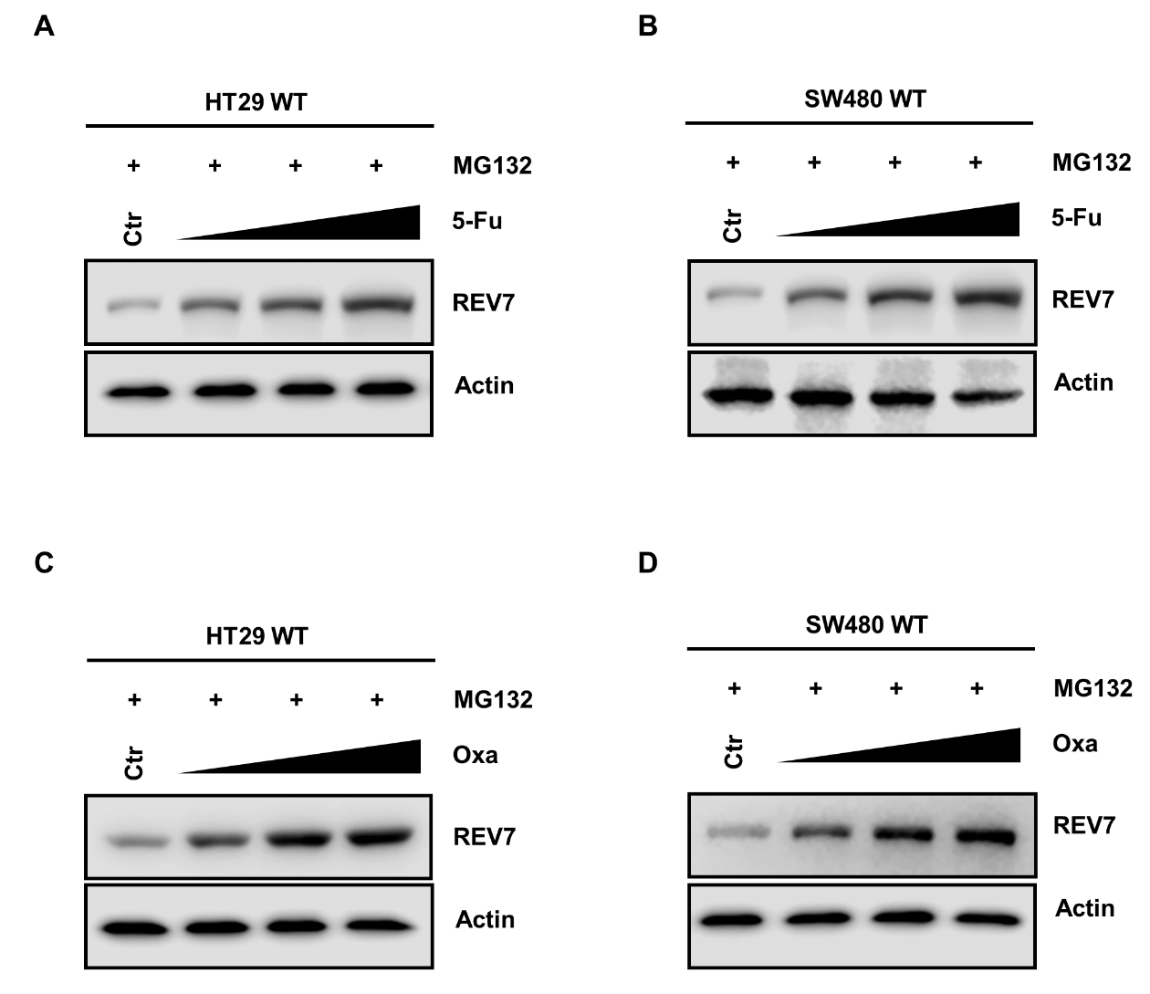


**Figure. S3 Increase of REV7 in response to 5-FU and oxaliplatin is not mediated by proteasome-mediated degradation**

**(A)** Western blot analysis of REV7 protein expression in response to 5-Fu in HT29 WT and **(B)** SW480 WT cells in the presence of MG132. Cells were treated with 0 µM, 0.5 µM, 1 µM and 2 µM of 5-Fu for 48 h, and subsequent 10 µM of MG132 for 4 h before harvesting for western blotting analysis. **(C)** Western blot analysis of REV7 protein expression in response to oxaliplatin in HT29 WT and (**D)** SW480 WT cells. Cells were treated with 0 µM, 2 µM, 4 µM and 8 µM of oxaliplatin for 48 h, and subsequent 10 µM of MG132 for 4 h before harvesting for western blotting analysis.


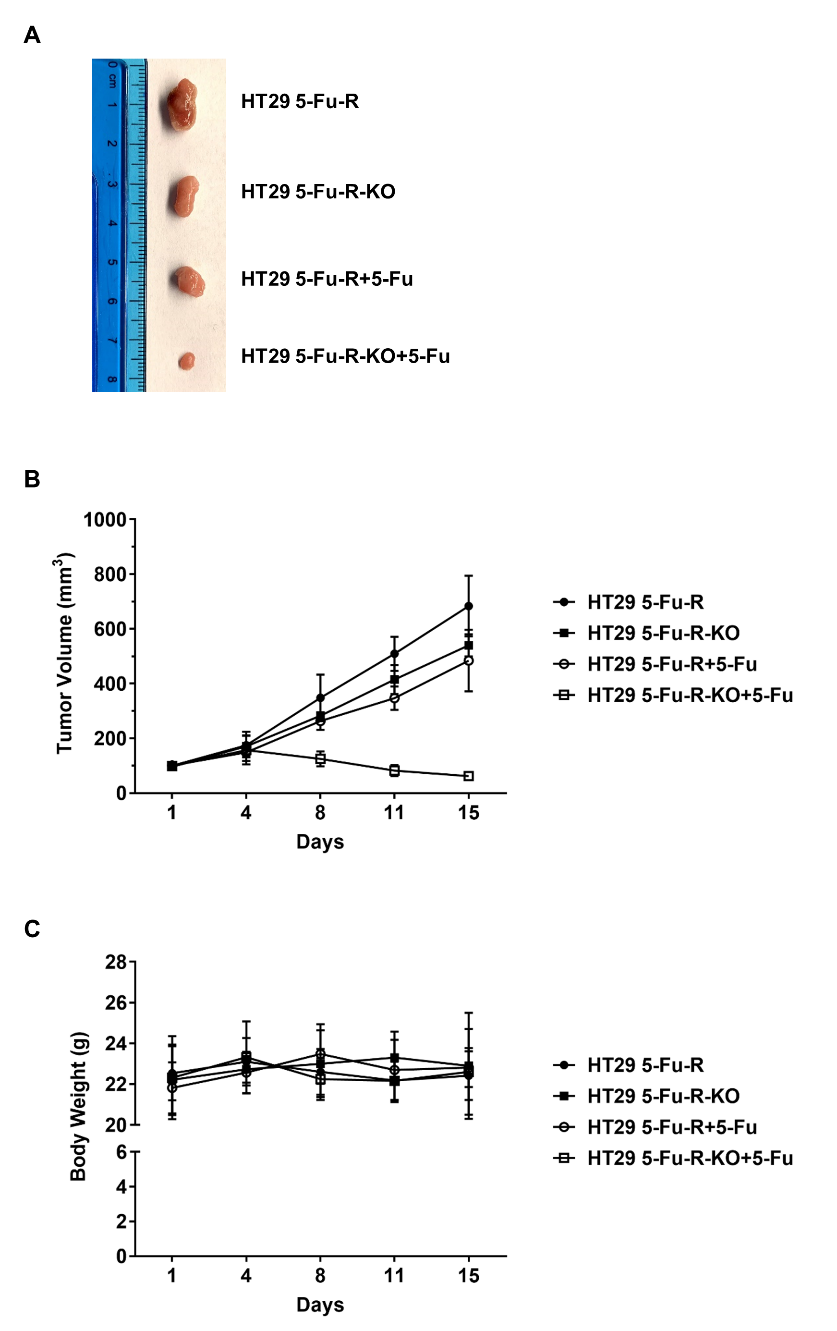


**Figure. S4 REV7-deficiency inhibited 5-Fu-resistant HT29 xenograft tumor growth *in vivo***

**(A)** Photograph of inoculated tumors excised at day 15. HT29 5-Fu-R and HT29 5-Fu-R-KO tumors with or without 5-Fu treatment were evaluated in this analysis. **(B)** Growth curve of inoculated tumors. Variation is indicated and presented as mean ± SEM. **(C)** Body weight of the mice measured post-drug treatment. No significant variation was detected. The statistical analysis of cell viability was calculated by using two-way ANOVA.
